# Biological Activity and Pharmacological Application of Pectic Polysaccharides: A Review

**DOI:** 10.3390/polym10121407

**Published:** 2018-12-19

**Authors:** Salima T. Minzanova, Vladimir F. Mironov, Daria M. Arkhipova, Anna V. Khabibullina, Lubov G. Mironova, Yulia M. Zakirova, Vasili A. Milyukov

**Affiliations:** 1 Arbuzov Institute of Organic and Physical Chemistry, FRC Kazan Scientific Center, Russian Academy of Sciences, Kazan 420088, Russia; minzanova@iopc.ru (S.T.M.); mironov@iopc.ru (V.F.M.); arkhipova@iopc.ru (D.M.A.); krayushkina@iopc.ru (A.V.K.); mironoval1963@gmail.com (L.G.M.); 2 Kazan (Volga region) Federal University, Kazan University, KFU, Kazan 420008, Russia; yulia_1505@mail.ru

**Keywords:** pectin, modified pectin, biological activity, drug delivery, pharmaceutical

## Abstract

Pectin is a polymer with a core of alternating *α*-1,4-linked d-galacturonic acid and α-1,2-l-rhamnose units, as well as a variety of neutral sugars such as arabinose, galactose, and lesser amounts of other sugars. Currently, native pectins have been compared to modified ones due to the development of natural medicines and health products. In this review, the results of a study of the bioactivity of pectic polysaccharides, including its various pharmacological applications, such as its immunoregulatory, anti-inflammatory, hypoglycemic, antibacterial, antioxidant and antitumor activities, have been summarized. The potential of pectins to contribute to the enhancement of drug delivery systems has been observed.

## 1. Introduction

In recent years, a surge of interest has been devoted to the research of natural polysaccharides (pectin in particular) for their wide range of biological properties and different applications in pharmacology [1,2]. The invention of quite simple as well as more advanced in vitro tests for influence on the immunity, accompanied with purification and characterization methods, are considered to contribute to these studies. Pectic polysaccharides have an immense potential in the healthcare, food, and cosmetic industries due to their therapeutic effects and relatively low toxicity.

According to specifications approved by the Food and Agriculture Organization, industrial pectins include at least 65% polygalacturonic acid, which is concomitant with different other conditions to satisfy the specification of E440 performing the function of the food additive. The most traditional raw materials that are used for the extraction of pectins are either apple pomace or citrus peels, which are by-products of juice manufacturing units. Both materials contain significant amounts of pectic substances, but with different chemical characteristics that make them suitable for specific applications [3]. Commercially, pectin is usually obtained from the residue of plant material after extracting the juice (apple or citrus peel) or sugar (sugar beet). The main trial applications of citrus or apple pectin are as gel-forming agents, thickeners, or stabilizers for acidic beverages. Extracts of sugar beet pectin are poor gel-forming agents, but show potential roles as emulsifiers [4]. The chemical modification of the structure is performed to increase its biological activity. Pectin is also capable of being used for drug delivery to the gastrointestinal tract in the covered form as gel beads, film, and matrix tablets.

**Figure 1:**
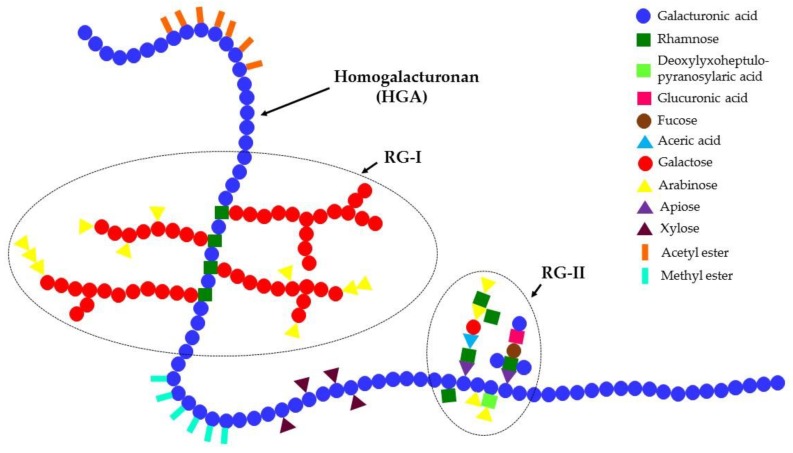
A scheme of the primary structure of pectins.

Pectins are biopolymers with multiple applications because of their structural diversity and complexity. Although pectins from different sources have some common structural characteristics, many aspects of the common structure change according to the species and the physiological stage of the plant. In addition, the application of pectin is determined by its chemical features, including galacturonic acid content, methoxyl content, and degree of acetylation [3]. Pectin is a polysaccharide with a core consisting of α-1,4-linked d-galacturonic acid and α-1,2-l-rhamnose units in turn, as well as a large number of neutral sugars, including arabinose, galactose, and lesser amounts of other sugars. Pectins have a linear anionic backbone with regions having no side chains known as “smooth regions”, and regions with non-ionic side chains known as “hairy regions” [1,5]. The structural classification of pectin includes: homogalacturonan (HG), rhamnogalacturonan I (RG-I), and substituted galacturonans such as rhamnogalacturonan II (RG-II) (Figure 1). All the main types of pectins are described in the article [1].

Currently, pectins have become an important part of the research and development of natural medicines and health products due to their wide availability. Pectins isolated from various plants as well as modified pectins are intensively observed. On one hand, this review covers the bioactivity of pectin polysaccharides, including their various pharmacological applications, i.e., their immunoregulatory, anti-inflammatory, hypoglycemic, antibacterial, antioxidant, and antitumor activities have been summarized, and their use as a carrier for drug delivery has been assessed. On the other hand, the research is focused on the relationship between the chemical structure and biological activity of pectins with the aim of expanding their use in medicine and pharmacology. 

## 2. Outline of Biological Activities of Pectic Polysaccharides

### 2.1. Immunoregulatory Activity

Immunomodulators are natural or synthetic substances that have a regulating effect on the immune system. By the nature of their influence on the immune system, they are divided into immunostimulating and immunosuppressive groups. The activity of immunostimulants is due to their ability to affect the metabolism of cells and tissues of the body, activating immunocompetent cells. Meanwhile, immunosuppressants are used to suppress the activity of lymphoid cells in inflammation, allergies, transplantation, and the treatment of autoimmune diseases. As immunostimulants, immunosuppressants are obtained from the tissues of animals and plants by biosynthesis, using genetic engineering and chemical synthesis methods [6]. Plant-derived polysaccharides, including pectins, can directly activate the immune function of macrophages, promote the production of cytokines, and therefore regulate the immune system on multiple levels. 

*Sambuci flos*, also known as elderflower, is conventionally used to cure different diseases that are connected with the immune system, such as for instance chill, influenza, or pyrexia. Giang Thanh Thi Ho et al. received 50% ethanol, 50 °C, and 100 °C aqueous extracts from *S. nigra* flowers that had intense fixing activity and macrophage stimulation effect [7]. The monosaccharide compositions of the fractions that were acquired as a result of anion exchange chromatography from 50% ethanol, 50 °C, and 100 °C water extracts have shown monosaccharides that are typical for pectins, including: arabinose, rhamnose, galacturonic acid, galactose, xylose, glucose, and mannose. The ratio between the monomers of different fractions, which varied considerably, was estimated, and a profound analysis of the original polysaccharide structures, as well as linkage analysis, was carried out. Structural alteration such as the removal of arabinose and ester impact the additional fixating and stimulating activities of macrophages. The nutritional significance of elderflower is growing due to the biologically active polysaccharides, which perhaps contribute to its high immunomodulating characteristics [8]. The isolated fraction showed no toxicity in the examined concentrations in 3-(4,5-dimethylthiazol-2-yl)-2,5-diphenyltetrazolium bromide (MTT) assay and the brine shrimp assay, registering no toxicity toward these cells. In vitro tests demonstrated that high-molecular RG-I-containing polysaccharides expose the greatest dose-dependent fixation of complement and macrophage-stimulating effects. The biological activity of these polysaccharides surpasses the biological effect of the corresponding polysaccharides after the equal procedure of isolation from the elderberries [9].

The immunomodulatory activities of the oligomer fractions are still observed in the studies of the enzymatic digestion of pectins, which also show the value of the backbone of pectins. The carbohydrate chain of pectins determines immunosuppressive activity. It was found that pectins containing more than 80% of galacturonic acid residues suppress the activity of macrophages and inhibit the delayed-type hypersensitivity reaction. In addition, the branched region of the pectin macromolecule mediates the stimulation of phagocytosis and the increased production of antibodies [10].

Lemon pectins were studied in the research by Vogt et al. [11]. Pectins with different degrees of methyl esterification, such as 30% (30DM), 56% (56DM), and 74% (74DM) that were purchased from CP Kelco were used to investigate the impact of backbone chain length and the methyl esterification degree of pectins on Toll-like receptors. The influence of pectin with various degrees of methyl esterification on intestinal barrier function in vitro was monitored, as pectins modulate the intestinal barrier, having important implications for human health [12]. The fractions that were digested demonstrated no activating capabilities. It has been found that the physical chemical properties of lemon pectin, susch as the degree of methyl esterification (DM; high DM pectins activating Toll-like receptors are much more pronounced than low DM pectins) and the extent of polymerization, can influence their immunostimulatory characteristics, and accordingly can be important when utilizing pectins to enhance immune status [11].

*Artemisia afra* is a plant that is used traditionally in South Africa against a variety of diseases such as internal worm infestation, cough, cold, fever, colic, headache, loss of appetite, pain in the ear, and malaria [13]. The research studies of the polysaccharides from *A. afra* demonstrate that its water-soluble fraction includes biologically active polysaccharides that exhibit immunomodulating effect. Prior to the extraction of the polysaccharides, the plant material was extracted with organic solvents to remove lipophilic and low molecular weight substances. The residue was then subjected to extraction with 50% ethanol in water, followed by extraction with water at 50 °C and finally water of 100 °C. All of the fractions were subjected to ultrafiltration with a cut-off of five kD. The fractions that were rich in carbohydrates and with similar migratory behavior were pooled, dialyzed, freeze-dried, and analyzed for bioactivity. All of the fractions have high contents of arabinose and galactose, indicating the presence of arabinogalactans, which are polysaccharides that are commonly present in pectins as side chains to the main core. The most striking feature among these is the relatively high amount of xylose present, which may have an impact on the bioactivity. It is known that the polysaccharides with the high bioactivity that is found in *Lessertia frutescence* also have the feature of xylose units linked to the galacturonic acid chain [13,14]. A main core of the polysaccharides consists of a rhamnogalacturonan backbone connected with longer chains of homogalacturonan. 

*Terminalia macroptera* Guill. and Perr. is a tree that originated in West Africa. The parts of this plant such as its leaves and stem bark have been used traditionally for medical purposes to cure cough and sores, hepatitis, and tuberculosis, as well as relieve pain. The roots are utilized against gonorrhea and different infectious diseases, including Helicobacter pylori-connected illnesses. The pre-extraction of the cells conducted twice over with 96% ethanol and 50% ethanol–water at 70 °C and following thorough the extraction with distilled water at 50 °C enabled extracting lipophilic and low molecular weight compounds. Then, the high molecular weight fractions isolated from the stem bark, root bark, and leaves of *T. macroptera* were filtrated, and then concentrated, dialyzed, and lyophilized. The three most abundant fractions possess the characteristics of pectic polysaccharides, consisting of hairy regions (RG-1) and smooth regions. The inhibition of lysis, which the test samples induced, was estimated, at 415 nm. A pectic polysaccharide with high activity produced by aerial parts of *Biophytum umbraculum* was utilized in the capacity of a positive control. Using these data, the calculations of the concentration of test samples producing a 50% inhibition of lysis (ICH_50_) was conducted by means of a dose-response graph. A low ICH_50_ degree means a soaring complement fixation activity. These studies revealed that the stem bark, leaves, and root bark of *T. macroptera* provide raw materials for fractions with bioactive polysaccharides [15].

*Lycium ruthenicum* Murr., serves as a source plant that is distributed mainly in China [16]. Traditional medicine utilized its fruit to cure hypertension, heart disease, and climacteric syndrome. Recently, some investigations indicated that *L. ruthenicum* revealed a variety of pharmacological properties, including antioxidation, antifatigue, and hypoglycemic activity [17]. The structural features of a pectin isolated from *L. ruthenicum* fruit have been investigated, and its immunomodulatory activity has been evaluated in vitro. The crude polysaccharides have been isolated by water extraction from the fruits of *L. ruthenicum* and purified. The *L. ruthenicum* pectin was obtained in a 0.086% yield based on the dried weight of the plant powder and contained 97.5% carbohydrate, according to the phenol sulfuric acid test. It was composed of galacturonic acid, rhamnose, arabinose, xylose, and galactose in the molar ratio of 4.7:1.0:2.2:0.5:1.2. Structural research exhibited that the backbone of pectins comprises α-1,4-linked HG rarely alternated with RG-I parts. As well, the Branford assay demonstrated that *L. ruthenicum* pectin contains 2.3% of protein. Glycine was the dominate amino acid, and other amino acids such as aspartic acid, glutamic acid, and serine are present in abundance. Nitric assay demonstrated the stimulation of macrophages due to the pectin activity, which can be estimated by the Griess method. It is reported that the *L. ruthenicum* polysaccharide inhibits the lipopolysaccharide induced nitric oxide (NO) production and the mRNA expression of inducible nitric oxide synthase (iNOS), and suppresses the levels of pro-inflammatory cytokines in lipopolysaccharide-stimulated macrophages [18]. 

Data on the immunoregulatory activity of pectins are summarized in Table 1.

### 2.2. Anti-Inflammatory Effect 

Inflammation has been considered as a main risk factor for different progressive illnesses in human beings, such as neurological disorders, cancer, metabolic diseases, and cardiovascular disease, and a primary strategy to prevent these diseases is to target the reduction of chronic inflammation [19]. The intake of dietary fibers such as plant cell wall polysaccharides enhances the efficiency of treatment of inflammatory processes. Much attention has been focused in recent years on pectins.

The pathology of inflammation is a complicated process triggered by microbial pathogens, such as viruses, bacteria, prion, and fungi [20,21]. Macrophages account for the first defense line of the human body. Lipopolysaccharides (LPS) are usually employed as a model for inflammation due to their ability to stimulate macrophages. Various inflammatory moderators are produced by macrophages that are influenced by inflammatory mediator nitric oxide and LPS, including cytokines such as tumor necrosis factor alpha (TNF-α) and interleukin-1β (IL-1β) [22]. The LPS-induced RAW 264.7 (cell lines mouse macrophages) is usually utilized as the anti-inflammation model detecting in vitro [23].

A water-extractable polysaccharide from starfruit (*Averrhoa carambola* L.) has been obtained [19]. Starfruit is an esculent tropic fruit that is commonly consumed as fruit juice, and has demonstrated a wide range of pharmacological activities. Polysaccharides from starfruit were segregated with boiling water, yielding the aqueous fraction, which after neutralization with acetic acid (HOAc), dialysis against tap water, and deionization with cation exchange resin resulted in some fractions. It was indicated as a substituted galacturonan by monosaccharide, methylation, gel permeation chromatography, and 2D NMR analyses. The effect of one of the fractions on the nocifensive behavior caused by the intraplantar injection of formalin in mice was estimated. This polysaccharide demonstrated antinociceptive and anti-inflammatory properties in a formalin model, suggesting that it has potential health benefits, and may be useful in therapeutic intervention for the management of inflammatory pain [19]. 

The structural features of pectic polysaccharides extracted from *Ulmus pumila* L. (PPU) were studied to evaluate the anti-inflammatory activity of PPU and selenized-PPU (Se-PPU) using RAW 264.7. It was shown that 2% and 4% of Se-PPU reduced LPS-induced NO production by inhibiting the protein expression of iNOS in RAW 264.7 cells [24].

*Suaeda fruticosa* (L.) Forssk is an annual halophytic plant shrub [25]. It has been demonstrated that the polysaccharides from *S. fruticosa* (SFP) can be a new source of an analgesic and anti-inflammatory agent or antioxidant with promising merits for healthy nutrition and therapy [26]. Isolated pectic polysaccharides from *S. fruticosa* have a significant potential in antioxidant activity, comprising lipid peroxidation, free radical scavenging, and decreased effect. What is more, SFP decreases carrageenan-induced edema within three to four hours. These effects of SFP can be explained by the considerable galacturonic acid content and rather low degree of esterified acidic groups and molecular weight [26].

The in vitro activities of acetylated pectin (OP) extracted from cacao pod husks, its fractionally de-esterified and de-acetylated form (MOP), and a commercial homogalacturonan on peritoneal macrophages in mice were studied. The results showed that a polysaccharide with better capacity than its native form to activate macrophages to a cytotoxic phenotype was produced after the deacetylation and de-esterification of pectin isolated from cacao pod husk. MOP seems to have potential medical application, which contributes to the reduced susceptibility to microbial infection, in addition to antitumor activity. The chemically modified biopolymers form an enhanced product with a new range of usage [27].

Popov et al. has studied the anti-inflammatory activity of citrus pectins in vivo after oral administration in mice [28]. Three models of inflammation were used: cytokine production by blood leukocytes in response to lipopolysaccharide, acetic acid-induced colitis, and endotoxin shock. The results of the study demonstrate that low methyl-esterified citrus pectin inhibits local and systemic inflammation, while pectin with a higher degree of esterification can inhibit intestinal inflammation. These findings verify that chemical features influence the physiological properties of pectins. The identification of a quantity of low methyl-esterified pectin chains in plant-based food would explain the wholesome qualities of the diet [28].

*Sedum dendroideum* is succulent plant that has been utilized to cure inflammatory diseases. Two pectic polysaccharides from the leaves of *S. dendroideum* were isolated [29]. The studies have shown the influence of pectic polysaccharides on the secretion of pro-inflammatory and anti-inflammatory cytokines by macrophages. If a pro-inflammatory agent was available, these polysaccharides demonstrate an anti-inflammatory activity, precluding the secretion of pro-inflammatory cytokines and supporting the secretion of an anti-inflammatory cytokine. From the findings, it was suggested that polysaccharides might cause the anti-inflammatory characteristics of *S. dendroideum* extracts, warranting the common application of this plant for curing inflammation disorders, ulcerations, and sores. This activity is also monitored for other polysaccharides that exhibit pro-inflammatory as well as anti-inflammatory effects, and as a result, could contribute an immunomodulatory function [27,30].

The inherent activation power of a pectic fraction extracted from sweet pepper fruits to cause cytokine secretion by THP-1 macrophages and regulate the lipopolysaccharide (LPS) pro-inflammatory response has been investigated. The structural modifications in the tested pectin were submitted, and its biological activities were compared with a native fraction to analyze the structure–function relationship. To sum up, native pectin and modified pectin at the highest concentration (300 μg/mL) possessed the inherent activation ability to control the TNF-α, IL-1β, and IL-10 secretion by THP-1 macrophages, but the chemical differences of these pectins facilitate an explicit influence on stimulation. On the other hand, due to LPS availability, the anti-inflammatory activity of native pectin occurred by decreasing the production of pro-inflammatory and soaring anti-inflammatory cytokines, concurrently reducing the TNF-α/IL-10 and IL-1β/IL-10 proportions. Native pectin might serve as an immunomodulator, in spite of the absence of a pure pro-inflammatory effect [31].

The profound analysis of the polysaccharides structures of Cabernet Franc (WCF), Cabernet Sauvignon (WCS), and Cabernet Sauvignon Blanc (WSB) wines were performed, and their anti-inflammatory properties were studied in vitro by Bezerra et al. According to their chemical composition, the polysaccharides of WCF, WCS and WSB comprised mannan, type II arabinogalactan, and traces of type II rhamnogalacturonan and type I rhamnogalacturonan. The conducted research shows that the pooled fractions of WCF, WCS, and WSB and fractions with extracted polysaccharides produced an anti-inflammatory effect on the LPS-induced RAW 264.7 mouse macrophage cells in vitro. These effects were promoted by reducing inflammatory cytokines (TNF-α and IL-1β) and the mediator inflammatory (NO) [20]. 

Yerba mate (YM) (*Ilex paraguariensis*) is a plant that originates from Uruguay, Paraguay, Brazil, and Argentina. In the study concerning yerba mate (*I. paraguariensis*), polysaccharide was extracted from leaves of this plant, and its chemical structure was defined as RG-I. An anti-inflammatory effect of RG-I could be verified by its capacity to reduce the tissue expression of inducible nitric oxide synthase (iNOS) and cyclooxygenase-2 (COX-2), and a possible subsidiary ability to treat sepsis [32]. Furthermore, the yerba mate RG-I contributed to suppress the gastric lesions stimulated by ethanol in rats [33].

It is reported that the *Lycium ruthenicum* Murr. polysaccharide (mentioned above, Section 2.1) reduces inflammation by means of restraining the Toll-like receptor 4 Nuclear factor-kappa-binding (TLR4/NF-_К_B) signaling pathway [16].

The study [34] demonstrated that the Firmicutes species *Eubacterium eligens* was an important, specialist degrader of diet-derived pectins in the human colon that produces a constitutive pectate lyase. The culture supernatant of this bacterium intensively promotes the synthesis of anti-inflammatory interleukin 10 (IL-10) in human peripheral blood mononuclear cells. It may possess powerful anti-inflammatory activities in vivo, and thus should be providently regarded as a possible next-generation probiotic. Its components could identify it as a structurally complex and chemically heterogeneous family of molecules pectins, thus providing useful fractional degradation products that may cause various prebiotic specificities and potential compared with native plant pectins. For instance, some *Faecalibacterium prausnitzii* strains, although restricted to reclaimed pectin, jointly with *E. eligens,* that have been grown on a purified pectic-oligosaccharides revealed compound could present a successful prebiotic approach for promoting these potentially beneficial Firmicutes species. Alternatively, a similar choice of pectin-utilizing species could be made among the Bacteroidetes, contributing to great interest in the consequences of developing a number of species within the genus. Recently, the research by Chung et al. [35] demonstrated the application of apple pectin as a sole carbohydrate substrate to human colonic microbial communities in anaerobic continuous culture with 16S rRNA amplicon sequencing to determine the most promising competitors. This presented stimulation of six various Bacteroides spp., whereas among the Firmicutes, just *E. eligens* were developed. Analysis of these findings demonstrated that *E. eligens* surged to an average of 15% of total 16S rRNA gene sequences in the pectin-fed communities, compared with only 1% in the fecal inoculum.

### 2.3. Hypoglycemic Effect 

Diabetes mellitus is a grave metabolic disease worldwide that affects millions of people. Over 90% of diabetic cases fall on the spectrum of Type 2 diabetes mellitus (T2DM), which is considered to be the dominating type of diabetes. Insulin resistance in T2DM causes impaired glucose tolerance and hyperglycemia. Diabetes is commonly treated with synthetic anti-diabetic agents that can provoke adverse side effects. In recent years, great attention has been paid to the anti-diabetic activity of dietary fiber [36].

Research [36] was conducted to study the anti-diabetic effect of citrus pectin in diabetic rats and the potential benefits of citrus pectin to produce anti-diabetic effects in cases of T2DM caused by a low-dose streptozotocin and a fat-laden diet. The first sign of metabolic diseases associated with T2DM proved to be insulin resistance. The oral glucose tolerance test can imitate the postprandial hyperglycemia state, presenting essential facts about the impact of insulin on glucose utilization [37]. Many tests have demonstrated that anti-diabetic polysaccharides effectively improve glucose tolerance [38,39]. Citrus pectin reduced fasting blood glucose levels, benefited hyperlipidemia, and refined hepatic glycogen content glucose tolerance in the diabetic rats. Citrus pectin modulated the expression of the basic proteins in the PI3K/Akt signaling pathway, which could have influenced the enhancement of insulin sensitivity in the diabetic rats, possibly by signifying the anti-diabetic effect of citrus pectin [40,41,42]. 

The fruits of *Abelmoschus esculentus* (L.) Moench, which is also famously known as okra, are widely spread in the Middle East, Asia, and Africa. Okra is traditionally applied in folk medicine due to its great number of acidic polysaccharides, which are also called pectin-like mucilages. The content of the RG-I domain was used to identify the pectic polysaccharide fraction extracted from the okra. It was shown that pectic polysaccharides suppress high hyperglycemic activity on STZ-induced diabetic mice by possibly retarding the peroxidation chain reaction, and could serve as powerful new therapeutics to diabetes [43].

*Panax ginseng* is the most widely used herb to cure diabetes. A large number of studies in animals and humans confirmed its hypoglycemic activity. The pectic polysaccharides were produced from red ginseng (GPR, steamed ginseng at 100 °C) white ginseng (GPW), and steamed ginseng (steamed ginseng at 120 °C, GPS) by means of conjunction of ion-exchange, water extraction, and gel permeation chromatographies. These pectic polysaccharides were segregated into two fractions, which were indicated as HG-rich and RG-I-rich pectins based on a combination of monosaccharide composition and ^13^C NMR analysis. The amount of Gal and Ara in GPS decline acutely with the processing temperature; nevertheless the galacturonic acid (GalA) content in GPS sharply surged in comparison with that in GPR and GPW. In vivo animal experiments demonstrated that GPS produced substantial antioxidant anti-hyperglycemic activities in alloxan-induced diabetic mice, and the influence increased with the processing temperature [44].

A homogalacturonan was produced from the fruits of *Ficus pumila* L. (FPLP) [45]. The administration of 100 mg kg^−1^ day^−1^ and 200 mg kg^−1^ day^−1^ of FPLP administration orally over four weeks notably enhanced the progressive deterioration of glucose homeostasis in mice, and the improvement of hyperglycemia was connected with increased hepatic glycogen content. In addition, the activation of the IRS-1/PI3K/Akt/GSK-3β/GS insulin signaling pathway and the modulation of basic hepatic glycogen metabolism-linked enzymes containing glucokinase, phosphoenolpyruvate carboxykinase, and glucose-6-phosphatase were indicated as the therapeutic mechanisms of FPLP. The study verified the theory that the hypoglycemic effect of FPLP could be determined by the enhancement of hepatic glycogen metabolism [45].

It should be expected that pectins, similar to many polysaccharides, will become a new class of hypoglycemic drugs.

### 2.4. Antibacterial Activity

In recent years, much attention has been devoted to the application of natural antimicrobial systems for the production of harmless and wholesome food [46]. In this connection, data on the antibacterial activity of pectins are of considerable interest, since they have a bactericidal effect on Gram-positive and Gram-negative microorganisms [6].

A simple green approach to synthesize Ag nanoparticles (NPs) using citrus pectin as the reducing and capping agent has been developed. Fourier transform infrared (FTIR) spectroscopy showed the pectin coated on the surface of Ag NPs. The Ag NPs exhibited good antibacterial activities toward Gram-negative *Escherichia coli* and Gram-positive *Staphylococcus aureus*. It has been established that the Ag NPs could potentially be antibacterial agents in biomedical applications. This work suggested a new strategy for the highly efficient use of citrus pectin in the future [47].

Biodegradable materials based on pectin, pectin-oleate, pectin-linoleate, and pectin palmitate were observed due to the antimicrobial effect against a number of bacterial strains, including *S. aureus* and *E. coli*. Pectin-linoleate and pectin-oleate demonstrated great capacity to suppress the growth of the selected microorganisms by 50–70%. They have the best antimicrobial activity against *S. aureus*. Water resistance and the protective properties of the polysaccharide were increased by chemically modified pectin, thus generating and deriving a bio-based material for new applications, such as for instance the safe way to package active food products [48].

Industrial samples of antimicrobial means without antiseptic medicines and antibiotics have been developed: namely, a stomatological bandage with prolonged staying in the extracted alveolar socket. The base of medicines that are under development is apple pectin. It is shown that the bacteriostatic activity of various pectin concentrations on the growth of broth cultures of staphylococci and streptococci, and on the creation of biofilms with the cultures of staphylococci and streptococci, proved to be more efficient in 1% solutions [49].

Pectin–cadmium sulfide nanocomposite (Pc/CSNC) was synthesized [50] at 60 °C in water phase through applying pectin as the connecting bargaining machine. Pc/CSNC showed a significant antibacterial effect against the culture of *E. coli*. One study [51] focused on the synthesis by the sol–gel method of biopolymer-based nanocomposite ion exchanger pectin zirconium (IV) selenotungstophosphate, which showed prospective antimicrobial activity against *S. aureus*, while the other [52] introduced the synthesis, characterization, and applications of a pectin-based zirconium (IV) silicophosphate nanocomposite (Pc/ZSPNC) ion exchanger. Pc/ZSPNC was revealed to have a considerable antimicrobial effect against *E. coli* and *S. aureus*. 

Chauhan et al. reported the fabrication of new pectin–GeO_2_ nanocomposites for possible electronic applications in new semiconducting materials. The suspension of a pectin–GeO_2_ nanocomposite has demonstrated high antibacterial activity against *E. coli* [53].

The study [54] was conducted to investigate and compare the antimicrobial effects of nanoemulsions comprising essential oils (EOs) (thyme, oregano, lemon grass) as the oil phase and high-methoxyl pectin solution (1% *w*/*v*) as the aqueous phase against *E. coli* and *Listeria innocua* during storage. To obtain the results, EO–pectin nanoemulsions were preserved at room temperature for 56 days. The assessment of the antimicrobial activity of tested nanoemulsions was performed at intervals in terms of *E. coli* and *L. innocua* population decline. Furthermore, the evaporation of the volatile fraction of EOs while in storage was analyzed and associated with the antimicrobial capability of nanoemulsions. The antimicrobial activity was not influenced by the type of applied EOs [54].

The surface of the cellulose nanofibrous mats was exploited to deposit negatively-charged pectin and positively charged lysozyme (LZ) alternately by the layer-by-layer (LBL) self-assembly technique. The nanofibrous mats covered by 10.5 LZ/pectin bilayers with LZ on the outmost layer revealed the most significant inhibitory effect toward *E. coli* and *S. aureus* according to the data of a bacterial inhibition test for cellulose mats and LBL-structured mats [55].

The antimicrobial effect of PGNaCo and PGNaNi was indicated at concentrations of 2.5%–10% against fungal cultures of *Candida albicans* and *Aspergillus niger* and *S. aureus*, *E. coli*, *Bacillus cereus*, and *Pseudomonas aeruginosa* by means of the replication method. Batches of the pectin metal complexes (0.25 g, 0.5 g, 0.75 g, and 1 g) were weighed and joined with meat-peptone agar (MPA) (10 mL) for bacteria and Sabouraud medium in agar (10 mL) for fungi. The composition effect demonstrated the antimicrobial activity of PGNaCo and PGNaNi (concentration 10%) against the test microorganisms. PGNaCo absolutely inhibited the development of the bacterial test strains and yeast-like fungus *C. albicans*, and partially that of mold fungus *A. niger*, while its concentration decreased from 10% to 7.5%. PGNaCo remained inactive against *A. niger* and *P. aeruginosa* in case of the further reduction of the concentration to 2.5% [56].

### 2.5. Antioxidant Activity 

Oxidation is vital to plenty of organisms that can generate energy to supply biological processes. In normal circumstances, free radicals govern cell growth, and suppress viruses and bacteria. Nevertheless, in large quantities and without regulation, the production of free radicals induced by oxygen cause cell damage, which renders the pathological progressions. The oxidative stress is associated with chronic obstructive pulmonary disease, asthma, diabetes, inflammation, cardiovascular diseases, and myocardial infarction. The synthetic antioxidants that are usually applied in manufacturing processes are considered to be cytotoxic. Natural polysaccharides are regarded as reliable antioxidants, possessing the ability to scavenge free radicals and surpass synthetic substances on the subject of health concerns [57,58,59].

The yerba mate (YM) (*I. paraguariensis*) powdered stems and leaves are utilized as additives for stimulant drinks [60] due to YM’s health virtues, including the antimicrobial and antioxidant properties [61] of the purified polysaccharide [62]. The structure of the YM purified polysaccharide is RG-I. The YM polysaccharide demonstrated valued antioxidant effect as assessed by 2,2-diphenyl-1-picrylhydrazyl (DPPH)-radical scavenging activity, 3-ethyl benzothiazoline-6-sulphonic acid (ABTS+)-radical scavenging activity, and hydroxyl activity to scavenge. The research determined that the YM polysaccharide antioxidant activities could be verified by various in vitro methods. Besides, the purified YM polysaccharide revealed significant antimicrobial activity against definite fungal and bacterial strains. The YM polysaccharide due to its antifungal effect could alleviate sepsis. Therefore, the YM polysaccharide may be recommended for pharmaceutical purposes and food production [62].

The rhizome of *Ligusticum chuanxiong* Hort has been actively utilized for centuries in folk Chinese medicine to cure cardiovascular diseases and migraines. It is known that polysaccharides isolated from *L. chuanxiong* are responsible for antitumor [63] and antioxidant [64] properties. By means of gel filtration and ion exchange chromatography, Chao Huang [65] obtained a high-purity polysaccharide from *L. chuanxiong* (LCP-II-I). Structural analysis showed that its long HG-1 segments alternated with RG-I segments, and side chains of arabinogalactan type I and arabinan. This LCP-II-I demonstrated effective protection against the oxidative action of the intestinal stress. It was shown that LCP-II-I treatment is a powerful way to safeguard SW480 cells against the oxidative stress triggered by H_2_O_2_.

*Castanea henryi* is a fruit that is used in dried conditions in conventional Chinese medicine [66]. In the study [67], a pectic polysaccharide extracted by hot water with further chromatography, and then dialyzed and lyophilized, allowed obtaining pure polysaccharide (CHIP3). Chaoyang Wei et al. evaluated the in vitro antioxidant activity of the substance and investigated the structure of the purified CHIP3 by means of FTIR. Five monosaccharides such as rhamnose, galacturonic acid, arabinose, mannose, and galactose are part of pectic polysaccharide CHIP3. Its primary structure consists of recurrent segments of an RG-I. In addition, CHIP3 demonstrated a high ferric decreasing-effects power with an 824.3 µM ferric reducing antioxidant power assay (FRAP) value at 1.0 mg/mL, which is a powerful scavenging degree for (ABTS+)-radical with EC 50 (246.85 µg/mL). Also, CHIP3 has a considerable restricting influence on HepG2 cells (IC 50 = 242.6 µg/mL) in the cytotoxicity testing in vitro. The research revealed CHIP3 to be a functional ingredient of nourishment that could possibly possess antioxidant properties [67].

Pomegranate (*Punica grantum* L.) growing in the regions of Iran and Balkan Peninsula has common application for medicine materials due to its outstanding antioxidant, antibacterial, antitumor, antiviral, immunoregulatory, and anti-inflammation activity [68]. By means of single-factor tests, the impact of the pH, ratio of liquid to solid, extraction temperature, enzymolysis time, and the dosage of enzymes on the production of pomegranate peel (PPP) extracted by pectinase were studied [69]. The in vitro antioxidant effects of PPP were ascertained by establishing its decreasing and scavenging influence on hydroxyl radicals, DPPH radicals, and superoxide anion radicals. Based on an analysis of the experimental results, it was shown that PPP can be applied as a significant antioxidant and embrace novel functional food applications.

Jackfruit (*Artocarpus heterophyllus*) with its large fruits is widely spread in the tropical and subtropical areas. The pulp is usually consumed fresh and processed by the inhabitants of China. Since almost 60% of the fruit is inedible, the large amount of non-utilized product caused a challenge, and inspired the investigation of techniques to utilize the waste. Pectin from jackfruit peel was obtained by means of various mineral acids and organic acids. In the research, microwave processes were performed alternating with an ultrasound method for the extraction of pectin from jackfruit peel. The monosaccharide composition of pectin was indicated to include galacturonic acid, glucose, arabinose, galactose, and rhamnose. The results confirm the promising ability of pectins from jackfruit peel to serve as natural antioxidants in food production [70].

Acerola (*Malpighia emarginata)* is a tropical fruit growing in the Caribbean islands and South America. Due to its significant vitamin C content, there is much demand for acerola in medicine. Its antioxidant activity determined the recommendation to apply it against different disorders induced by oxidative stress [71,72]. Molecular weight, homogeneity, total uronic acid, and monosaccharide composition analyses were carried out [73,74]. The study included mainly a pectic polysaccharide and biological model [75]. The research showed the capacity of pectin to protect the cell against the cytotoxicity that was generated by H_2_O_2_ by means of a decrease of intracellular reactive oxygen species levels.

In the study [76], an effective microwave-assisted extraction technique was applied to produce pectic polysaccharide (TPPs) from tangerine peel with the yield of 20%. As a result of purification by chromatography pectic polysaccharide, TPPs-2-1 was obtained. TPPs-2-1 comprised generally GalA, Rha, Ara, Gal, Man, Glc. TPPs-2-1 possessed α-glycosidic bonds, and had a typical IR spectra that was characteristic of pectic polysaccharides. Moreover, TPPs-2-1 had a significant FRAP value, as well as high radical scavenging effects against •OH and •DPPH; however, it also had low scavenging indices against •O^2−^. The properties of TPPs-2-1 was influenced by its molecular weight, as well as the composition of monosaccharides and methods of extraction. The results verified the capacity of TPPs-2-1 to be antioxidant, as well as its capacity for application in pharmacology and as an ingredient for functional food [76].

Alperujo, a by-product extracted from the manufacture of olive oil, was treated by a new method of gentle heating at 50–80 °C over one to two hours, and was then carried out in a three-phase centrifugation system that produced alperujo oil, olive oil cake, and aqueous by-products. Firstly, the pectic substances were obtained hydrothermally by the treatment of alperujo at 160 °C for 30 min, 45 min, or 60 min [77]. The pectins that were isolated in these conditions possessed a significant degree of esterification and a low molecular weight. The properties of the pectic substances obtained from olive mill wastewater were studied in comparison with commercial pectins from apple and citrus [78]. The purification procedure decreased the proportion of GalA compared to the neutral sugar amount in the pectic material, which predominantly consisted of arabinose (Ara) (32% and 34%), GalA (24% and 18%), galactose (Gal) (17% and 16%), glucose (Glc) (9% and 16%) and rhamnose (Rha) (7% and 11%). The polysaccharides extracted from alperujo demonstrate metabolic activity and may be applied to regulate cholesterol metabolism and control diabetes in vitro. The antioxidant activity, bile–acid binding, and glucose-retardation capability of the polysaccharides were estimated in vitro. The raw and purified polysaccharides, including two functions of dietary fiber and antioxidants, can produce beneficial effects in the colon, contributing to the prevention of chronic diseases, including bowel cancer.

In the report [79], pectin was obtained from dehydrated Jerusalem artichoke (*Helianthus tuberosus*) residues with 5% moisture in weight. The impact of pH, extraction temperature, and liquid-to-solid ratio on pectin output were examined. In addition, the associations between pectin properties such as antioxidant effects, surface structures, and drying conditions were investigated. The DPPH radical-scavenging activities of Jerusalem artichoke pectin dried by the three different methods were indicated with the slightly modified technique. The results of the work reveal the noncritical role of drying methods in terms of the structure of Jerusalem artichoke. Assessments on the antioxidant effects of pectins treated by various methods indicated that spray-dried pectin and apple pectin possess higher activity than pectin after vacuum oven and vacuum freeze desiccation. The reduction activities of pectin are connected with the presence of hydroxyl groups and electron transfer from pectin (ROH or RO–) to DPPH. It was investigated that the pectin of Jerusalem artichoke can be an available antioxidant source of plant origin that could be used in the preparation of food [79].

Alcoholic and aqueous extracts from various of fruits were produced; then, the antioxidant activity of every sample were characterized, and the main bioactive components in them were estimated. The film characteristics after the addition of fruit extracts with stronger antioxidant effects than pectin-based films were studied. Pectin films comprising fruit extracts were assessed in relation to their phytochemical contents, ultraviolet light transmission, and antioxidant activity for 90 days of shelf life storage. The chemical, mechanical, and physical properties, as well as the antioxidant stability of the films were characterized. The results of the research of phenol compounds, including the content of anthocyanin, vitamin C, carotenoids, and antioxidant activity of the extracts from pequi, acerola, cashew apple, papaya, and strawberry were obtained. The insertion of extracts into the pectin films contributed to the higher antioxidant activity of these materials, and the pectin film with acerola extract possessed the best antioxidant capacity; thus, it is another potential material in coatings for different food applications as well as for antioxidant films [80]. 

The study [81] was aimed at developing an edible pectin film saturated with essential oil, which can surge the antioxidant status and decrease the bacterial development in freshly cut peach. Cinnamon leaves were used as the natural source. Food-grade pectin dissolved in distilled water formed the film-producing solution, which was stirred for 15 min. After the addition of the plasticizer glycerol, the solution was restirred for 15 min. After preparation, the films were placed in storage in desiccators, at 4 °C of nitrogen atmosphere, protected from light, and analyzed *in vitro* for a week. The total antioxidant activity was identified by means of the DPPH method that Molyneux presented. The edible pectin film with cinnamon leaf oil revealed an appreciable antioxidant capacity, which could be beneficial for both extending the shelf-life of food and for the human diet [81].

Ogutu and Mua investigated the influence of ultrasound effect, including its power, duty cycle, and time, on sweet potato pectin’s structure, molecular weight, antioxidant activity, and neutral sugar composition. Sonication increased the antioxidant activity of 200-W and 400-W sonicated pectin, obtaining more significant oxygen radical absorbance capacity (ORAC) and ferric reducing antioxidant power assay (FRAP) values, with considerable pectin content providing inherently high antioxidant activity than native and 100-W treated pectin. The ORAC value of 400-W sonicated pectin quintupled what native pectin possessed, when the FRAP amount was three times as high as native pectin. This is due to the fact that the ultrasonic treatment reduces the molecular weight of pectin, while polydispersity did not reveal an obvious trend as determined by incidental pectin scission, soaring the duty cycle from 20% to 80%, and contributing to a threefold decrease in the pectin’s molecular weight. A sharp rise of galacturonic acid content from 72.0% ± 1.2% in native pectin to between 85.0% ± 3.2% and 92.0% ± 2.7% was influenced by the surged sonication power. Meanwhile, the degree of methoxylation declined from 12.0% ± 3.0% to between 5.25%–6.28%. Nevertheless, ultrasound did not change the primary structure of the pectin, as verified by FTIR and High-Performance Anion-Exchange Chromatography results. It was demonstrated that ultrasound provides an eco-friendly and effective method for pectin transformation, and powerful pectin products regarding antioxidant content can be developed [82].

The influence of the structural characteristics of citrus pectin—particularly the degree of methylation (DM) and the degree of blocking (DBabs)—on the oxidative and physicochemical stability of linseed/sunflower oil emulsions (LSSO), which were earlier treated with a synthetic emulsion stabilizer, were investigated. The DM of pectin did not contribute to the physicochemical stability of the LSSO emulsions, but obviously affected their oxidative stability. Low DM pectin (≤33%) revealed more significant lipid antioxidant capacity in comparison with high or intermediate DM pectin (≥58%). The pectin lipid antioxidant effect may be caused by its ability to chelate pro-oxidant Fe^2+^. The research presumes that citrus pectin can perform as a natural alternative to artificial antioxidants. Furthermore, pectin additives in edible emulsions can contribute to produce clean food products with antioxidant properties, since pectin can demonstrate different functionalities [83].

The meat industry uses synthetic antioxidants that are inferior to natural safe antioxidants such as pectin, such as butylated hydroxyanisole (BHA) and butylated hydroxytoluene (BHT) [84,85]. The small misshapen, bruised, and/or overripened Japanese plums (*Prunus salicina*) that are discarded can be an alternative carbon source for chemical commodities (i.e. food additives), adding value to the raw materials. Basanta et al. [86] beneficiated the chicken meat product with the freeze-dried fiber microparticles (MPCs) of Japanese plum extracted separately from the peel and pulp. The meat product constitutes a worthwhile matrix to assess the application of plum peel and pulp MPCs as antioxidant food additives. The obtained protein–carbohydrate products possess functional properties.

The antioxidant and anti-inflammatory activity of honokiol (HNK) and modified citrus pectin (MCP) have been investigated in vitro in the report [87]. The dependence of the antioxidant activity of HNK and MCP on their doses was shown, wherein MCP revealed a significantly higher antioxidant capacity. The MCP:HNK (9:1) combination showed a synergistic effect on antioxidant activity. Further study into this composition is reasonable, owing to the significant combination effect to inhibit cyclooxygenase-II activity, nuclear factor-kappa B activity, and tumor necrosis-α synthesis in mouse monocytes [87].

Flax (*Linum usitatissimum*) has been a significant crop plant for human beings. The authors [88] compared the polysaccharide composition and phenolic compound contents of transgenic plants and control plants, as well as the mechanical properties of flax fibers, in order to establish the usefulness of this transgenic plant for textile and pharmaceutical production. The contents of such polysaccharides as hemicellulose, cellulose, and pectin in transgenic flax were in abundance, and a decreased amount of lignin was detected. The inhibition of •DPPH radicals in the cell wall fraction was estimated. The transgenic plants revealed a higher degree of antioxidant activity in comparison with the control plants, which proved their possible application in biomedicine [88].

It should be taken into account that pectins may provide auxiliary functions for active antioxidant component. Results of the studies are shown in Table 2.

The antioxidant activity of methyl cellulose (MC) was compared with those for high methoxyl pectin (HMP) in edible films, putting an emphasis on stability of ascorbic acid (AA). The findings verified that the stability of AA hydrolytic surged and the non-enzymatic browning decreased due to the hydrophobicity of MC. HMP and MC could form physical blends. The 50:50 HMP:MC films revealed high antioxidant activity, protecting walnut oil. The polymeric film microstructure influenced the stability of the AA and antioxidant properties [5].

An ester form of retinol and palmitic acid, presented as retinyl palmitate (RP), possesses active anti-aging properties that have been utilized to cure and prevent wrinkles; it is an essential ingredient of anti-aging cosmetics. The antioxidant activity of pectin and other polysaccharides for the development of cosmetics products with antioxidant activity similar to RP has been studied. The effects of the composition parameters of pectin on the degradation profiles of RP were researched. Furthermore, the effect of pectin on the stability of RP was considered as a time function. In the group of the polysaccharides that were tested, pectin displayed a significant antioxidative effect, which was analyzed by DPPH radical-scavenging method. The antioxidative activity of pectin seems to be caused by the presence of the hydroxyl group. The lower concentration of RP (0.01%, *v*/*v*) impacted the higher stabilizing effect of the pectin. The authors considered that pectin could be applied as a cosmeceutical component for RP stabilization [89].

Four polyphenol-conjugated pectins (catechin, quercetin, rutin, and hesperidin) have been synthesized as a result of epichlorohydrin conjugation reactions between polyphenol molecules and pectin in aqueous conditions. The conjugates demonstrate better solubility in water in comparison with neat pectin and polyphenols. The obtained new conjugates were discovered with satisfied stability to keep a common structure–antioxidant property relationship, and metal ion-binding capacity was achieved. These pectin conjugates with cross-linking and antioxidant characteristics possess new applications that have been usefully employed in the pharmaceutical, food, biomedicine, and cosmetics fields [90].

### 2.6. Antitumor Activity 

#### 2.6.1. Antitumor Activity of Pectin

So far, cancer remains a global disease, and the investigation of effective treatment is a great challenge. Metastasis cause the majority of cancer-related deaths in spite of obvious enhancement in gene therapy, surgery, chemotherapy, immunotherapy, and radiotherapy [91]. The therapy is complicated by the drug resistance of tumor cells, and serious side effects after chemotherapeutics have become the burning issue. Many in vitro and in vivo studies concerning the antitumor activity of native and modified pectin revealed a decrease of adhesion and cell proliferation, as well as the induction of apoptosis and migration [3].

Maxwell et al. have assessed pectin from different sources (potato, sugar beet, larch, and citrus) for effects against colon cancer cells [92]. RG-I extracts of potato pectin lowered the proliferation of colon cancer cells by the alteration of dose. Intercellular adhesion molecule-1 (ICAM-1) gene expression that had been diminished by RG-I and ICAM1 expression and influenced by siRNA contributed to the reduction of cell proliferation, developing a new mechanism for the powerful antitumor effect of pectin. HG component and neutral sugar side-chains could exert significant action on the anti-proliferative activity. The impact of four potato pectins on human colon cancer cells and the probable factors that were responsible for the effects were studied individually [93]. The research data verified the association between the RG-I domain and the anti-cancer effects of this pectin.

Sugar beet pectin extracts presenting various structures of pectin showed high anti-proliferative action against colon cancer cells. The alkali treatment of pectin surged the antitumor activity of sugar beet pectin due to an apoptosis promotion. Alkali treatment decreased the DE and increased the ratio of RG-I to HG. Thus, the RG-I/HG backbone could possess biological activity, because the influence on cells remains, even after the elimination of the side-chains of neutral sugars. However, the galactan and arabinan side-chains improve bioactivity [94].

The pectic polysaccharide from apple can induce the death of cancer cells death and suppress the growth of tumors in vivo, as Delphi and Sepehri described. They compared the activity of apple pectin in the form of pectic acid and modified pectin toward cancer metastasis. The results verified that pectic acid revealed apoptotic activity, and was able to inhibit breast tumor cells growth in vivo. These studies established the in vivo reduction of tumor growth and size due to the stimulation of apoptosis and supposed suppressing angiogenesis, wherein animal body weight wasn’t changed considerably [95].

*Lonicera japonica* is the conventional remedy for curing sores, infection diseases, exopathogenic wind-heat, wounds, and furuncles. Homogenous RG-I polysaccharides were obtained and purified from the flowers of *L. japonica*, and their structure and influence on the activity of pancreatic cells were investigated. The polysaccharide was described as an RG-I backbone. Its monosaccharide composition presented galacturonic acid, rhamnose, arabinose, and galactose. The polysaccharide demonstrated high inhibition regarding the proliferation of pancreatic cancer cell BxPC-3 and PANC-1, while a weak cytotoxicity of the live cells was observed. The study suggests that pectic polysaccharide from flowers of *L. japonica* could be a powerful novel medicine for pancreatic cancer [96].

Three polysaccharides (PLE-0, PLE-I, and PLE-II) were isolated from persimmon leaves, and their antitumor and antimetastatic activities were analyzed. Both PLE-0 and PLE-II considerably amplified natural killer (NK) cells, which caused cytotoxicity toward lymphoma tumor cells and suppressed the lung metastasis that was promoted by colon carcinoma cells. PLE-II failed to stop tumor metastasis due to the depletion of NK cells, showing that NK cells play a major role in regulating the antimetastatic effect of PLE-II. Besides PLE-0 being the crude polysaccharide, it also induces anti-cancer and immunomodulatory activity [97].

The *Hippophae rhamnoides* L. from the Tibetan plateau has been traditionally utilized as medicinal food. Water-soluble polysaccharide (HRWP-A) was isolated and separated by Hailiang Wang et al. from *H. rhamnoides* berry, occurring as a mixture of glucan and HG, with high antifatigue activity [98]. Structural analysis, as well as the immunological and antitumor activity assays, were conducted thoroughly [99] to describe the structural features, and thus investigate the possible pharmaceutical application of polysaccharides from the *H. rhamnoides* berry. An antitumor effect study indicated the capacity of HRWP-A to inhibit the Lewis lung carcinoma development in tumor-bearing mice. Further testing verified that immunostimulating properties can influence the antitumor effect of HRWP-A due to improvements in the lymphocyte proliferation soaring macrophage activities and providing NK cell activity and cytotoxicity T-lymphocyte (CTL) in tumor-bearing mice.

The anti-cancer effect of sweet potato pectin on colon cancer cells after ultrasonic modification was assessed in order to investigate its possible use in anti-cancer therapy. It was shown that sonicated pectin exhibited higher cell anti-proliferation compared to native pectin. Overall, sonication improved pectin anticancer capacity, and could offer an effective, affordable, and easily scalable means of biofunctional pectin production for the pharmaceutical sector. Moreover, sonication could be applied in the modification of other polymers to achieve certain characteristics [100]. 

It was shown that citrus pectin after modification by means of heat treatment (60 min, 123 °C, pressure 17.2–21.7 psi) caused cell death in two cancer cell lines. The death of induced cells stood in marked contrast to classical apoptosis due to the absence of DNA cleavage. The results demonstrated the activity of autophagy. The unique case of autophagy has been observed in cells that were incubated with available pectin in a modified form. This autophagy activation seems to be shielding, for adenocarcinomic human alveolar basal epithelial cells, due to the high cytotoxicity of modified pectin, which is mediated by its inhibition with 3-methyladenine. The research verified the capacity of modified pectin to contribute to effective chemotherapy [101].

The physicochemical properties of extracted pectic polysaccharide from apple pomace by hot compressed water were investigated and compared with commercial pectin. It has been shown that the free radical-scavenging capacity and inhibitory activity on colon adenocarcinoma cells increased in the extracted pectic polysaccharide [102].

One of the directions in antitumor research is the inhibition of galectin-3 (a lectin associated with cancer progression and metastasis). In general terms, pectins containing a more significant amount of neutral sugar possess higher bioactivity that is induced by the hypothesis in which galactan side-chains on pectin may bind to and suppress the pro-metastatic protein galectin-3, causing the inhibition of cancer cell aggregation, proliferation, metastasis, and adhesion. pH-modified citrus pectin was fractionated by using a combination of salting out, ion-exchange, and gel-permeation chromatography in order to identify the active components. Functionally, modified citrus pectin followed by elution with aqueous 0.25 M of NaCl contributed to suppressing galectin-3-mediated agglutination, and the RG-I-rich pectins with 1,4-linked β-D-galactan side-chains demonstrated higher activity in comparison with the others. Furthermore, the research findings indicated that RG-I-type and HG-type pectins can perform mutually, due to their effects being considerably decreased upon fractionation [103].

It is known that the structure of pectiс polysaccharides (including size, composition, and branching pattern) can vary, depending on their source. The pectin structure is reported to influence its bioactivity. The structure modification of pectin can be thermal, chemical, or enzymatic. The last one occurs during the natural processes of fruit ripening by the coordinated action of endogenous pectinolytic enzymes, including the fractional depolymerization of pectin to a smaller size with reduced branching, while an interim stage of fruit ripening takes place. It was shown that papaya pectin obtained at various ripening stages contributes to the death of cancer cells. Ripening processes were followed by the depolymerization of pectic polysaccharides, comprising arabinogalactans, RGs, HGs, and their modified forms. The pectin being extracted from the intermediate ripening stage of papaya is definitely reduce the cancer cell viability. Besides, culture wound closing is delayed in three forms of immortalized cancer cell lines. The data can be explained by the peculiarities of papaya pectins on the third day of harvesting, such as the disturbed collaboration between the extracellular matrix proteins and cancer cells, which improves cell detachment and develops apoptosis/necroptosis. The presence and the branch of the arabinogalactan type-II structure impacts the anti-cancer effect of papaya pectin [104].

Data on the antitumor activity of pectins are summarized in Table 3.

#### 2.6.2. Antitumor Activity of Pectin Conjugates

Curcumin, a natural polyphenolic phytoconstituent extracted from *Curcuma longa* L., Zingiberaceae (turmeric), has been applied in the biomedical field [105]. Curcumin produces a variety of pharmacological activities, including anti-inflammatory, antioxidant, anti-hyperlipidemic, and anti-cancer properties, as well as wound healing. However, due to its utterly low aqueous solubility, the bioavailability and stability of its application in medicine is limited [106]. The study investigated the inhibitory activity of pectin–curcumin (PEC-CCM) conjugates toward cancer cells [107]. The anti-cancer effect of PEC-CCM conjugates was estimated by MTT [3-(4,5-dimethylthiazol-2-yl)-2,5-diphenyltetrazolium bromide] test containing breast and hepatic cervical cancer cell lines, as well as a human normal kidney cell line. The comparison with free curcumin revealed that the curcumin of PEC-CCM conjugates obtained higher inhibitory activity against cancer cells and reduced cytotoxicity for normal cells. The improvement in the conjugates’ cytotoxicity to cancer cells was probably because of the increased water solubility and relative stability of PEC-CCM conjugates. Simultaneously, the good biocompatibility of pectin could contribute to the considerable reduction of the cytotoxicity of curcumin against normal cells, as this conjugate significantly reduced the cytotoxicity of curcumin to normal cells. Futhermore, conjugated curcumin displayed more distinct cytotoxicity and anti-oxidant effect than free curcumin. The research suggested the possible existence of a synergistic effect between curcumin in the conjugates and pectin.

Since selenium (Se) is of vital importance for human health, it can be a powerful tool either in cancer prevention or in cancer treatment, especially when it is combined with other antitumor substances. W. Chen et al. obtained a selenized derivative of pectic polysaccharide (sCPP1b) from Wen-Codonopsis pilosula, and assessed its antitumor activity in comparison with an original pectic polysaccharide (CPP1b). It was shown that CPP1b and sCPP1b had the capacity to inhibit cell migration, alter cell morphology, cause cell apoptosis, and prevent the cell cycle. CPP1b and sCPP1b promoted apoptosis on adenocarcinomic human alveolar basal epithelial cells by the upregulation of caspase-3 and Bax, and downregulation of B-cell lymphoma 2 proteins. The study revealed the higher anticancer effect of sCPP1b in comparison with that of CPP1b [108].

*Artemisia sphaerocephala* Krasch occurs commonly in China, and has been applied in traditional medicine to cure asthma, hepatitis, and rheumatoid arthritis [109]. *A. sphaerocephala* polysaccharide was isolated and purified; the carbohydrate, protein, and uronic acid contents were 90.2%, 2.4%, and 16.3%, respectively. In vitro antitumor assays of selenized *A. sphaerocephala* polysaccharides (SeASPs) showed higher anti-proliferative activity toward three tumor cell lines (lung adenocarcinoma cells, hepatocellular carcinoma (HepG2) cells, and cervical squamous carcinoma (Hela) cells) by a dose-related method. Selenylation was supposed to considerably improve the antitumor effects of polysaccharide derivatives in vitro. Sulfated *A. sphaerocephala* polysaccharides (ASPs) were prepared, and the antitumor activity was evaluated in tumor cells and Hepatoma 22 tumor-bearing mice. Within in vitro experiments, ASPs significantly inhibited the growth of HepG2 and Hela cells. Moreover, no direct cytotoxicity against mouse fibroblast normal cells was observed in vitro. After oral administration for 12 days, the tumor growth was significantly suppressed by ASPs. The results of tumor histological morphology and cell cycle analysis showed that ASPs could arrest H22 cells at the S phase and promote cell apoptosis. Additionally, immunohistochemical analysis displayed that ASPs influenced the downregulation of mutant p53 protein expression in a dose-dependent manner [110]. Besides, the conducted research verified the capacity of selenylation and sulfation to enhance the antioxidant characteristics of polysaccharide derivatives [111].

Gaikwad et al. designed pectin-poly(vinyl pyrrolidone) [PVP]-based curcumin particulates to increase the antitumor activity of curcumin. Pectin–PVP-based curcumin particulates (PECTIN–PVP CUR, CP1–CP4) were produced by spray-drying methods in various ratios. The angiolytic activity and MTT investigation of PECTIN-PVP CUR revealed significant antitumor effects in comparison with curcumin alone. Studies on the effect of curcumin and CP3 on lung cancer cells A549, demonstrated dose-dependent cytotoxicity. It is proved that curcumin showed less cytotoxicity (% growth inhibition) compared to particulates (CP3) [112].

A self-assembled nanoparticles platform based on pectin–dihydroartemisinin conjugates for the co-delivery of anti-cancer drugs was developed by Liu et al. [113]. A pectin-based nanocarrier was designed as a combined simultaneous multiple-cargo consisting of the hydrophobic drugs dihydroartemisinin and 10-hydroxycamptothecin to be delivered to tumor sites. The pectin–dihydroartemisinin/hydrooxycampothecin nanoparticles (PDC-H NPs) with a small particle size of ~70 nm were produced. In vitro tests displayed that the PDC-H NPs revealed a higher cellular uptake, greater cell apoptosis induction, and cell-viability inhibition capacity in contrast with dihydroartemisinin and 10-hydroxycamptothecin. PDC-H NPs could actively capture mammary carcinoma cells by confocal microscopy imaging [113].

The nanocomposites can be used in various fields that encompass different biomedical applications, including cancer treatment, targeted drug delivery, molecular imaging, tissue engineering, and biosensors. Nanocomposite preparations using different noble metals have been incorporated in targeted drug delivery applications due to their known antimicrobial and anticancer properties. Different proteins and polysaccharides have been utilized to produce nanomaterials inducing antitumor effects [114]. 

Silver nanoparticles were conjugated with pectin by Nwakwasi et al. to estimate the antitumor properties of nanocomposites against the canine fibroblast tumor (A-72) cell line. It was shown that the pectin-tagged silver nanocomposite exhibited a very significant cytotoxic effect on the A-72 cancer cell line when compared with the standard anti-cancer drug (Fluorouracil/5-FU) by attaining a maximum inhibition percentage of 85.88%, which is comparable to the 88.88% of fluorouracil/5-FU. This clearly indicates the antitumor efficacy of the synthesized nanocomposite when conjugated with pectin, and thus proved to be a potential anticancer drug [115].

In the study [116], pectin, an anionic polysaccharide isolated from *Musa paradisiaca*, was employed for the synthesis of gold nanoparticles. The anti-cancer potential of prepared nanoparticles was evaluated in mammary adenocarcinoma. There was an apoptosis induction mediated by gold nanoparticles (p-GNPs), and a decrease in the viability of treated cancer cells (MCF-7 and MDA-MB-231) was observed. It was found that 50% of MCF-7 and MDA-MB-231 cells lose their viability at eight μg/mL and two μg/mL of p-GNPs at 48 h, respectively.

The study [117] presented a non-complicated green strategy for synthesizing ZnNPs with natural polysaccharides such as chitosan, citrus pectin, alginate, and the aqueous extract of fermented fenugreek by *Pleurotus ostreatus*, using gamma irradiation as a new reducing and fixating agent. The synthesized ZnNPs explored dispersibility, with an average size of 46–53 nm, and induced anti-cancer properties, inhibiting Ehrlich Ascites Carcinoma and human colon adenocarcinoma, as well as demonstrating a bactericidal effect against Gram-positive and Gram-negative bacteria due to the smaller size.

Intervention studies involving animals or humans, and other studies requiring ethical approval, must list the authority that provided the approval, and the corresponding ethical approval code. 

## 3. Pectin as Carriers for Drug Delivery

The successful treatment of different diseases is a direct result of effective carrier systems for the delivery of therapeutic agents or imaging agents. The bioavailability, safety, and effectiveness are the essential requirements for perfect carriers. The nontoxicity, targeting ability to a particular site, stability, and non-immunogenicity are also very crucial. The incorporation of a drug into a polymer matrix enables prolonging its action and optimizing pharmacokinetics. Moreover, the use of polymer film as an immobilization matrix facilitates the targeted delivery of the drug. The review [118] focused on advanced carriers based on natural biomacromolecules for drug delivery. Biomacromolecules exploded as carriers; they comprise proteins (transferrin, albumin, collagen, lipoproteins, keratin, and silk fibroin) and polysaccharides (hyaluronic acid, chitosan, heparin, cyclodextrin, and pectin). Due to the remarkable properties for pharmacology, such as ease of dissolution in common environments, mucus, and the capacity to produce gels in acids, pectins are of promising fundamental and applied interest [119].

The research on drug–polymer interaction is absolutely vital, and covers the issue of the modulated release of the drug from the formulation. The study of domperidone (DOM) was performed to investigate releasing an in vitro drug delivery system consisting of a floating complicated structure, with pectin proving to be an ideal medium for delivering DOM. In the study, DOM floating beads were composed by means of an extrusion congealing method. The effect of presented formulations was estimated, and the activity of different types of compositions was observed. The major advantages of the system could comprise significant encapsulation efficiency, simplicity of fabrication, and continued and persistent drug release upwards of 12 hours [120]. 

The group of authors used the methods of ionotropic gelation to develop a new hydrolyzed polyacrylamide-graft-sodium alginate (PAAmg-SA) and diclofenac sodium (DS)-loaded interpenetrating polymer network (IPN) beads of pectin. This research concerning the hydrolyzed PAAm-g-SA copolymer synthesized by free radical polymerization under the nitrogen atmosphere was continued with alkaline hydrolysis. The results of the investigation verified that hydrolyzed PAAm-g-SA and pectin cross-linked with Al^3+^ and glutaraldehyde could form an optimal matrix material for the production of IPN beads to support the sustained release of DS. A drug-release study was carried out in acidic solution (pH 1.2) and alkaline solution (pH 6.8 phosphate buffer) in vitro [121].

The aim of the study [122] was to analyze powder-based compositions for the nasal administration of tacrine hydrochloride. The anti-Alzheimer drug was encapsulated in mucoadhesive microparticles based on chitosan/pectin polyelectrolyte complexes. Microparticles were produced by methods of spray-drying followed by lyophilization and direct spray-drying, and their morphology, size, and physicochemical characteristics were investigated. The results of the study demonstrated the possible application of chitosan/pectin polyelectrolyte complexes in the composition of mucoadhesive microparticles, obtaining various functional features. The water uptake and tacrine hydrochloride permeation were influenced by the chitosan/pectin molar ratio. Drug-release studies were performed in vitro using Vision Classic 6 Dissolution Tester (Hanson, CA, USA) [122].

Hintzen et al. reported a new synthesis to obtain a completely S-protected thiomer. The unmodified pectin was compared with thiomer in vitro. The S-protected thiomer was produced by coupling pectin and an S-protected ligand, followed with the connection of the ligand l-cysteine and 2-mercaptonicotinic acid by a disulfide exchange reaction. The surplus of l-cysteine was deleted by ion exchange chromatography, followed by coupling of the S-protected ligand to pectin as a model polymer backbone. The final product data revealed that the S-protection enhanced the stability of the thiomer in solution at high pH, as well as developed the cohesive and adhesive characteristics of the thiomer. Respectively, the S-protection seems to have the capacity to enhance the beneficial properties of thiomers for potential usage in mucoadhesive drug delivery systems and the development of liquid thiomer-based formulations [123]. 

The aim of the research [124] was to compare and characterize commercial low-methoxyl pectin and a pectinmethylesterase charge-modified citrus pectin (MP 38), and indicate the possible application of loaded hydrogel beads for colonic drug delivery. Then studies were conducted to determine the indomethacin-loading ratio, as well as various distributions of charge and CaCl_2_ concentrations. Commercial high-methoxyl citrus pectin was not applied, revealing insufficient charge to cause ionic interaction with Ca^2+^. The pectin type was highly considerable for encapsulation efficiency and in vitro release assay. MP 38 hydrogel composed with 300 mM of CaCl_2_ and a ratio of 0.5% indomethacin demonstrated the significant entrapment activity. The investigation proved the enhanced encapsulation efficiency of orally administered drugs for a colon-targeted drug delivery due to charge modification of pectin [124].

Research on the efficiency of targeted functionalized nanoparticles in determined cells was conducted on the ligand–receptor interaction between glycoprotein CD44 and hyaluronan (HA) as a model. The cytotoxicity of the hyaluronan–cisplatin conjugate nanoparticles (HCNPs) was estimated. HCNPs were molded in Eudragit S100-coated pectinate/alginate microbeads (PAMs) by means of a polyelectrolyte multilayer-coating technique and an electrospray method in aqueous solution. Therefore, they conjugated HA–cisplatin into nanoparticles, and in simulated gastrointestinal conditions, the release pattern of the HCNPs from Eudragit S100-coated PAMs was evaluated in vitro. The obtained data confirmed the potential function of Eudragit S100-coated HCNP–PAMs as carriers for colon-specific drug delivery [125].

A chitosan–pectin polyelectrolyte complex was prepared by the simple interaction of positively-charged chitosan with negatively-charged pectin. The testing of the colon delivery of enteric-coated pills by means of the PEC was conducted. The optimum composition, including 1.1% PEC and 3% coating, revealed the most significant swelling and release regarding the alkaline pH mechanism, which was verified by an ex vivo study with rat caeca content, and occurred as a result of the degradation caused by microbial enzymes [126]. 

Another investigation [127] revealed the suitability of pectin–chitosan hydrogels for use as drug carriers, as well as a control mechanism for release patterns. In vitro drug-release studies verified that a lower percentage of pectin caused slower release rates due to the smaller mesh size that was produced by intense interactions between the polyelectrolytes. The release decelerated if the aggregate polymer concentration increased. Negatively-charged pectin interacting with chitosan, which is positively charged, generates an electrostatic effect, lessening the hydrogel network’s mesh size. The ionic strength of the phosphate buffer saline solutions did not affect the hydrogels’ characteristics. Drug-release studies showed a persistent release of three model drugs (mesalamine, curcumin, and progesterone) over 24 hours in a physiological environment [127].

The potential applications of pectin with DE9 as a formulation with a degree of esterification (DE) of 9%, and DE31 as a formulation with a degree of esterification of 31%, were demonstrated as sustaining the floating of paracetamol with further in situ gelation in the stomach of gastric acidity-controlled rats with variety of pH (1.2–6.8) [128]. A substantial distinction regarding the in vivo gelling characteristics of the pectin DE9 and DE31 patterns was revealed by testing the stomach contents of gastric acidity-controlled rats at high pH intervals, following the oral administration of 1.5% (*w*/*v*) pectin sols. The significant gastric acidity influenced the decrease of the DE31 effectiveness, in comparison with compositions produced from pectin with DE9, and the stomach pH surged to 5.5 [128].

The propolis by-product extract, which was combined with pectin to obtain spray-dried microparticles, including the dipeptide *l*-alanyl-* l*-glutamine as promoting systems of neutrophils, was described. The thermal constancy of the formulation was determined, and pectin, *l*-alanyl-* l*-glutamine, and propolis by-product extracts were spread over the matrix. The microparticles released the drug in vitro for 24 h, which was regulated by swelling and diffusion. The drug-loaded compositions demonstrated a valuable stimulating impact on neutrophils. These structures could strengthen the activity of the immune cells [129].

Polyelectrolyte complexes based on pectin are widely applied in drug delivery. The review [1] in great detail showed that pectin-based compositions could be utilized as bone replacement material or in tissue regeneration. Pectin/protein-based emulsions surpassed protein-based emulsions in stability. It is reported that pectin-based polymers were discovered as having a novel potential to create highly technological materials with enhanced characteristics. Casein–pectin as a multiple-unit drug delivery system was designed for the colonic drug delivery of two model drugs with different solubilities: acetaminophen (PCT) and indomethacin (IND) [130]. The effect of chemical cross-linking and the drying method (spouted or spray-drying) on in vitro drug release, microcapsule digestibility, and structural integrity was investigated. The results showed that the drying method that was selected affected the product quality, and that the successful spouted bed-drying of microparticles depended on the mechanical strength of the particles and the chemical characteristics of the drug and the polymer. It was also shown that сasein–pectin microparticles seemed powerful to extend indomethacin release, and the casein–pectin interaction did not prevent an enzymatic attack on the polysaccharide. Acetaminophen-loaded microparticles were successfully processed by both the spouted-bed and spray-drying methods, but the casein–pectin formulation must be improved if water-soluble drugs are to be delivered more efficiently. 

The study [131] aimed to investigate the possibilities of complex formations between citrus pectin (CP) with antibiotic ceftriaxone (Ctx) and of the formation of model composite films. Polymer films that were capable of controlled ceftriaxone release have been prepared based on the citrus pectin mixture with polyvinyl alcohol (PVA). Kinetics of Ctx release into the surrounding solution was determined by measuring the solution absorbance at 245 nm. The decisive role of the supramolecular structure of the polymeric matrix on the films’ transport properties has been demonstrated. 

The calcium gel capsules were formed by various pectins as low-methyl pectin, amidated pectin, and high-methyl pectin. The gel capsules with live probiotic *L. casei* were coated with chitosan. The impact of pectin concentration on the production of gel beads was firstly evaluated. The extrusion of these solutions into 0.15 M of CaCl_2_ determined the different shapes of the formation of gel beads, and was related to the type of pectin that was used and the concentration of its solution. In vitro experiments were also carried on with the encapsulated probiotic bacteria to indicate the protective effect of chitosan coating for the transit of live bacteria through the gastrointestinal tract [132]. 

Newly oxidized pectin–gelatin–nanosilver (OP-Gel-NS) nanohydrocolloids and ciprofloxacin hydrochloride that were incorporated into the OP-Gel to produce OP-Gel-Cipro dressings were developed by Tummalapalli et al. To study the release of the drug from OP-Gel-Cipro dressings in vitro, a Franz diffusion cell was utilized. The histological testing indicated that OP-Gel-NS and OP-Gel-Cipro dressings displayed a high hydrophilicity and sustained antimicrobial activity. As mentioned above, nanoparticles support cell growth and proliferation, and contributed to the fast soar cicatrizing ability due to the hydrophilicity and antimicrobial nature [133].

A new strategy for the preparation of cross-linked protein polysaccharide complex nanoparticles with unique stability in gastrointestinal environments and the prospective power of future utilization as agents for the oral administration of lipophilic nutrients was developed. In the study, protein–polysaccharide complex nanoparticles were produced from NaCas, zein, and three polysaccharides (carboxymethyl cellulose (CMC), pectin, and gum arabic) via a pH and heating-induced electrostatic adsorption process. Curcumin was investigated as a model lipophilic nutrient to test the encapsulation and delivery usage. The study demonstrated that various types of polysaccharides exerted different influences on the complexation. The particle size of pectin–NaCas–zein nanoparticles was sharply decreased from 472 nm to 247 nm, while CMC–NaCas–zein (CCZ) and gum arabic–NaCas–zein nanoparticles remained much the same post-heating. Consequently, the polysaccharides influence on the physicochemical properties of complex nanoparticles such as the kinetic release, drying, colloidal stability, curcumin encapsulation efficiency. The cross-linked CCZ nanoparticles exhibited high stability in simulated gastrointestinal conditions, and redispersibility in water [134]. 

The research concerning the combination of pectin with an insufficient charge density alginate of substantial charge gravity was conducted to form the coat around zein nanoparticles. The substitution of 30% of pectin with alginate considerably enhanced the aggregation stability at an increased ionic strength at pH 5 to 7. The curcumin-loaded core–shell nanoparticles demonstrated more excellent radical disposing effects than curcumin dissolved in ethanol. Consequently, the curcumin loaded core–shell nanoparticles as functional food and liquors could double as pharmaceutical products for dietary purposes [135].

The synthesis of pectin–silica gels to control the release of the drug in the gastrointestinal tract (GIT) by means of low-methoxyl and high-methoxyl pectins and tetraethoxysilane (TEOS) as a precursor was depicted. The combination pectin–TEOS and pectin–Ca–TEOS displayed the beads with the highest resistance. The hardness of the pectin silica beads influenced the drug release of the beads in simulated GIT, reaching up to 80%. Pectin–Ca–TEOS beads occurred to be stronger under mechanical compression, and in solutions simulated the intestinal environment drug release slower than that pectin–TEOS beads. FTIR demonstrated that hydrogen and covalent bonds between silica and pectin macromolecules were formed. By combination of the sol-gel and ionotropic gelation methods, various pectin-silica beads with mesalazine were synthesized. The in vitro drug release of pectin–silica beads is dependent on the beads’ hardness. It was concluded that the pectin–silica matrixes are promising regarding the elaboration of the controlled release of drug formulations [136].

The antimicrobial activity of nisin-loaded pectin–inulin particles as the main objective of the study was compared with nisin-loaded pectin particles. Inulin contributes to surge the effectiveness of nisin loading beside nisin–pectin particles. The antimicrobial activity of nisin-loaded pectin–inulin particles was influenced by the extent of pectin esterification. The significant activity corresponds to a low or zero degree of pectin esterification, while the particles of a more considerable degree of pectin esterification by contrast decreased the antimicrobial activity [137].

The study of a controlled-release gel matrix was conducted for powerful antibiotic enrofloxacin against Gram-positive and Gram-negative bacteria and keratinase with a base on a cryogel of polyvinyl alcohol–pectin (PVA-pectin) to cure eschars and wounds. Keratinase was immobilized onto PVA-pectin cryogel patches, testing pectins with different esterification degrees as well as with pectins of various biopolymer concentrations to look for an optimum antibiotic and enzymatic release. Examined formulations of cryogels were found to be environment-friendly for enzymes and provide enzymatic activity. Such a system for the immobilization of enzymes is versatile regarding the absorbance of proteins while retaining their biological activity. The in vitro enrofloxacin release from PVA-pectin cryogel indicated the ability of keratinase to retard the antibiotic release. Thereby, it causes prolonged and controlled release [138].

An emergent ecologically-friendly method to synthesize hydroxyapatite (HAP) nanoparticles utilizes naturally renewable pectin as a matrix. The impact of pectin concentration on the morphology, particle size, crystalline behavior, and purity of the as-synthesized HAP nanoparticles has been determined. The soaking of in vitro synthesized nanoparticles in simulated body fluid for several days revealed that the HAP nanoparticles that had been treated with the prickly pear peels-derived pectin surpassed the nanoparticles obtained without pectins in antimicrobial activity against *S. aureus*, *E. coli,* and *C. albicans* bacterial pathogenic strains. It can be applied as a powerful biological antimicrobial-covering material in orthopaedy and dental medicine [139].

The aim of the research [140] was to create active composite films based on pectin, and confirm the influence of clove ether as an additive on the mechanical and blocking properties of the produced films. Antimicrobial films were evolved by including various ranks of clove bud essential oil (0.5%, 1.0%, and 1.5%) into the citrus pectin with the aim of modifying the functional characteristics of the films. The microbiological evaluation by an agar disc-diffusion test verified the antimicrobial effectiveness of pectin films against Gram-positive *L. monocytogenes* and *S. aureus*. The study demonstrated that clove oil, combined with citrus pectin, is promising for producing antimicrobial edible pectin films to be utilized as coatings in the food industry [140].

Doxorubicin (DOX), a DNA intercalating agent, has been used as an effective chemotherapeutic for many types of solid tumors in breast, lung, ovarian, prostate, and bladder. However, its use is severely limited by its side effects. In order to decrease them, a drug delivery system was proposed. By using pectin (PEC) as a carrier material and doxorubicin (DOX) as a model drug, the blank PEC nanoparticles (PEC-NPs) and the DOX-loading PEC nanoparticles (DOX-PEC-NPs) were prepared. The PEC-NPs promoted the anticancer activity of DOX by enhancing the intracellular drug uptake in the cancer cells. In vitro, the DOX-PEC-NPs demonstrated a stronger inhibitory rate in the test cancer cells than the DOX solution [141]. 

It was suggested that pectic polysaccharides can be an effective organic matrix for the delivery of the macroelements and and microelements that are necessary for normal metabolism. Novel water-soluble pectin complexes with Fe^2+^, Zn^2+^, Mg^2+^, Ca^2+^, Co^2+^, Cu^2+^, and Mn^2+^ based on pectic polysaccharides were synthesized, and the animal assays were conducted on rats. The anti-anemic effects of new pectin complexes with (1) Na and Fe, and (2) Na, Ca, and Fe were studied in white rats with post-hemorrhagic anemia. As a result of the action of the complexes, an improvement in the state of the animals and the prevention of erythropoiesis disorders were observed. The anti-anemic effect of the complexes was manifested in doses equivalent to 25% or 50% of the daily iron norm, which was recommended in the treatment of iron deficiency anemia with preparations based on ferrous sulfate [142].

Another strategy for the preparation of pectin-based hydrogels treated with a cross-linking agent such as Fe^2+^ has been proposed [143]. The virtue of using Fe^2+^ is to protect active components from the oxidation that is produced as a result of complexation with pectin, and form the hydrogel performing cross–linking activity. Pectin macromolecules are obviously protected from stomach acidity in the course of digestion, while the macromolecules are cross-linked.

## 4. Conclusions

Over the past decade, a large number of published papers have dealt with various important bioactivities of pectic polysaccharides. Our review provides some theoretical recommendations for the further research of pectin according to the relationship between natural sources, chemical structures, biological activities, and practical applications in the food industry as well as pharmacology and different branches of medicine. The analysis of studies described here shows great insight into the structural requirements for pectin bioactivity and lack of detailed investigations at different levels for understanding the bioactive roles of pectins.

Pectins of different structures provide significant immunomodulatory properties. However, further research studies are necessary in order to verify the immune activity in vivo and determine how the mechanisms of pectins affect macrophages and other immunocytes for safe clinical applications in the future.

The studies of the antimicrobial properties of pectins show a general tendency for the development of nanocomposites and nanoemulsions on their base. Both of them exhibit noticeable inhibitory effects, mainly against Gram-negative Escherichia coli and Gram-positive Staphylococcus aureus. It is worthwhile to go more deeply into the study of existing composites, because the development of natural antimicrobials is a promising perspective.

The analysis of the reviewed studies shows that pectic polysaccharides from various sources (plant materials, food industry waste, and modified pectins) demonstrate antioxidant activity. It was also shown that pectins of diverse chemical structure (HG, RG-I, RG-II) exhibit antioxidant properties.

The hypoglycemic activity of pectins is useful for the development of new types of low-toxicity antidiabetic agents. Drugs based on dietary and medicinal plants don’t cause side effects, which makes pectins promising for further research.

The study of the anti-inflammatory properties of pectic polysaccharides is also given great attention in the literature, because pectins have a great potential for anti-inflammatory multi-purpose therapy. However, the development of multi-purpose drugs is still in its infancy.

Another fast-growing field of pectin useful practical application is anti-cancer therapy, which is due to safety of pectin and its derivatives. However, the lack of research on pectic polysaccharide protection mechanisms and clinical trials has limited the application of pectin in the field of medicine thus far.

This review shows that different types of pectin may be used for drug delivery. Some of the physicochemical properties of pectin include: the ability of dissolution in basic environments, muco-adhesiveness, degradation stability against proteases and amylases of the upper gastrointestinal tract, and ease of forming gels in acid environments. Such properties contribute to its ability to target various drug delivery formulations such as beads, microspheres, microparticles, and pellets.

To sum up, pectins are very interesting, and are a promising subject for further research in pharmacology and medicine application, largely due to their considerable availability from renewable sources and the non-toxic effect. They show high biological activity; however, there is still a long way to go into pectin action mechanism research. The current review can provide a guide to the development of new non-toxic industrial drugs on the basis of the ability of pectins to cure different diseases and enhance the state of health.

## Figures and Tables

**Table 1:** Immunoregulatory activity of pectins. HG: homogalacturonan, RG-1: rhamnogalacturonan I.

Pectin source	Extraction method	Pectin type	Monosaccharide composition	Bioactivity test	Reference
*Sambuci flos*	(1) 50% EtOH, 50 °C; (2) water, 100 °C	RG-I	Ara, Rha, GalA, Gal, Xyl, Glc, Man	*in vitro*	[7]
Lemon pectins (Kelco)	-	-	-	*in vitro*	[11]
*Artemisia afra*	(1) 50% EtOH; (2) water, 50 °C; (3) water, 100 °C	HG, RG with side chains consisting of arabinogalactan type II	Ara, Rha, Fuc, Xyl, Man, Glc, GlcA, Gal, GalA, 4-O-Me-GlcA	*in vitro*	[13]
*Terminalia macroptera* Guill. and Perr.	(1) 96% EtOH; (2) 50% EtOH, 70 °C; (3) dist. water, 50 °C	RG-I	Ara, Rha, Fuc, Xyl, Man, Glc, GlcA, Gal, GalA	*in vitro, in vivo*	[15]
*Lycium ruthenicum* Murr.	water	HG, RG-I	GalA, Rha, Ara, Xyl, Gal	*in vitro*	[18]

**Table 2:** The auxiliary function of pectin for active antioxidant components.

Antioxidant	Pectin function	Reference
Ascorbic acid	Stabilizer	[5]
Retinyl palmitate	Stabilizer	[89]
Polyphenols	Water solubility increase	[90]

**Table 3:** Antitumor activity of pectins. Gal: galactose, NK: natural killer.

Pectin source	Modification of pectin	Pectin type	Mechanism of action	Cancer cell lines	Reference
Potato pectin (Megazyme International Ireland Ltd.)	-	RG-I	inhibition of the proliferation of HT-29 cells and induction of significant G2/M cell cycle arrest	colon cancer cells	[93]
Sugar beet pectin (Kelco)	Alkali treatment	RG-I, HG	induction of apoptosis	colon cancer cells	[94]
Apple pectin (Fluca)	-	Pectic acid	induction of apoptosis, inhibition of cell growth (*p* < 0.001), reduction of cell attachment, fragmented chromatin, membrane blebbing	breast tumor cells	[95]
*Lonicera japonica* flowers	-	RG-I with galacturonic acid, rhamnose, arabinose, and galactose	inhibition of cancer cells’ growth	pancreatic cancer cell BxPC-3 and PANC-1	[96]
Persimmon leaves	-	RG-I, RG-II	augmentation of NK cell-mediated cytotoxicity against lymphoma tumor cells, inhibition of lung metastasis	lymphoma tumor cells, lung metastasis promoted by colon carcinoma cells	[97]
*Hippophae rhamnoides* L. berry	-	High-methoxyl HG	enhancing the lymphocyte proliferation, augmentation of the macrophage activities, promoting NK cell activity and CTL cytotoxicity	lung carcinoma	[99]
Sweet potato pectin	Ultrasonic modification	HG	apoptosis induction	colon cancer cells	[100]
Citrus pectin (Sigma)	heat treatment (60 min, 123 °C, pressure 17.2–21.7 psi)	HG	induction of cancer cell death	adenocarcinoma cells, liver hepatocellular carcinoma	[101]
Apple pomace (Yantai Andre Pectin Co. Ltd., Yantai, China)	Extraction by hot-compressed water	HG	inhibition of cancer cells growth	colon adenocarcinoma cells	[102]
citrus pectin (Sigma)	modified with aqueous 0.25 M NaCl	RG-I, HG	inhibition of Gal-3-mediated agglutination	-	[103]
papaya pectin	-	arabinogalactan, RG, HG	inhibition of the interaction of extracellular matrix proteins laminin, collagen IV, and fibronectin in cancer cells	colon cancer cell lines, prostate cancer cell	[104]

## Abbreviations

HG, homogalacturonan; RG-I, rhamnogalacturonan I; RG-II, rhamnogalacturonan II; GalA, galacturonic acid; Rha, rhamnose; Ara, arabinose; Gal, galactose; Man, mannose; Glc, glucose; CP, citrus pectin; PEC, pectin; DM, methyl esterification; HMP, high methoxyl pectin; DE, degrees of esterification; MOP, de-esterified and de-acetylated form of pectin; MC, methyl cellulose; CMC, carboxymethyl cellulose; HA, hyaluronan; PVA, polyvinyl alcohol; IND, indomethacin; Ctx, ceftriaxone; DOX, doxorubicin; STZ, streptozotocin; NO, nitric oxide; LPS, lipopolysaccharide; AE, alcoholic extract; WE, aqueous extract; iNOS, inducible nitric oxide synthase; COX-2, cyclooxygenase-2; TNF-α, tumor necrosis factor alpha; IL-1β, interleukin-1β; T2DM, type 2 diabetes mellitus; LZ, lysozyme; DPPH, 2,2-diphenyl-1-picrylhydrazyl; ABTS+, 3-ethyl benzothiazoline-6-sulphonic acid; ICAM-1, intercellular adhesion molecule-1; MTD, maximum tolerated dose; GIT, gastrointestinal tract.
